# Cone-Beam Computed Tomography Evaluation of Maxillary First and Second Molars in Iranian Population: A Morphological Study

**Published:** 2014-07-05

**Authors:** Armita Rouhani, Ali Bagherpour, Majid Akbari, Majid Azizi, Amirhosein Nejat, Neda Naghavi

**Affiliations:** a *Dental Research Center, Faculty of Dentistry, Mashhad University of Medical Sciences, Mashhad, Iran; *; b *Postgraduate Student of Orthodontics, Mashhad University of Medical Sciences, Mashhad, Iran;*; c *General Practitioner, Private Practice*

**Keywords:** Canal Configuration, CBCT, Cone-Beam Computed Tomography, Iranian Population, Maxillary Molar

## Abstract

**Introduction: **The aim of this *in vitro* study was to identify the root and canal morphology of maxillary first and second molars in Iranian population by taking and analyzing cone-beam computed tomography (CBCT) scans. **Methods and Materials:** Extracted maxillary first (*n*=125) and second (*n*=125) molars were collected from native Iranians and scanned by using a CBCT scanner. The number of roots and configuration of root canal system were classified according to Vertucci’s classification. **Results:** Two (1.6%) maxillary first and two (1.6%) maxillary second molars had four roots. Prevalence of root fusion was 2.4% and 8.8% in maxillary first and second molars, respectively. The most common canal morphology in the mesiobuccal roots of three-rooted first and second molars was type I (46.4% and 80.8%, respectively), followed by type VI (17.6%) in first molars. The predominant morphology of distobuccal and palatal roots in first and second molars was type I. Additional canal types were also identified.** Conclusion:** Profound knowledge of anatomic variations is necessary prior to cleaning and obturation of the root canal system. The most common discovered root morphology was three separate roots in both tooth types. The greatest variation in canal anatomy was discovered in the MB canals of both the first and second molars.

## Introduction

Proper three-dimensional (3D) cleaning and obturation of the root canal system is a prerequisite for the successful endodontic treatment. Therefore, determining the configuration of the root canal anatomy is necessary [1]. Morphological variations in root canal anatomy due to ethnicity have been reported in many studies; therefore, identifying the root canal anatomy of different ethnic populations is required for successful endodontic treatment [2-4].

To identify variations in root canal morphology, various techniques have been proposed: conventional and modified tooth staining and clearing [4-8], conventional and digital radiography [9-11], contrast media radiography [12, 13] and computed tomography (CT) [14].

Cone-beam computed tomography (CBCT) scanning, *aka* dental CT scan, has definitive advantages over conventional medical CT scans. CBCT uses an extra-oral imaging scanner to produce 3D images of the maxillofacial skeleton with a considerably low radiation dose compared to conventional CT scanning [15, 16]. Furthermore, CBCT scan has a resolution which is almost eight times higher than that of medical CT scans [17]. A CBCT scan captures diagnostic data in a collective volume instead of thin slices and all the voxels are isotropic; therefore, objects can be accurately measured in different directions. In contrast, a conventional medical CT scan cannot be equally accurate in different planes due to its anisotropic voxels [18]. Recent technologic advances have made CBCT scans available as a feasible option for the private dental practice [17].

The ability of CBCT to reduce or eliminate superimposition of surrounding structures makes it superior to conventional periapical films [15]. Therefore, CBCT is the best imaging technique for evaluation and identification of root canal morphology. Apart from its diagnostic accuracy and feasibility, CBCT does not damage the tooth structure as most *in vitro* studies. Moreover, it saves time during laboratory assessment of root canal morphology compared to staining and clearing techniques. No published article is available regarding the evaluation of the morphology of maxillary first and second molars with CBCT technique in Iranian population in the English literature. The aim of the current *in vitro* study was to identify external morphology of the roots and analyze the root canal configuration in maxillary first and second molars in Iranian population by means of CBCT technique.

.

**Figure 1:**
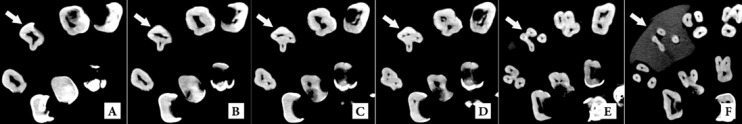
Maxillary first molar with fusion of two roots (DB and DP) in the axial section: *A)* Pulp chamber floor; *B)* Two canals (B and P); *C)* Three canals (MB, DB, P) with MB being separated; *D)* Four canals (MB, DB, MP, DP); *E)* While MB is separated, three roots are still fused; *F)* DB and DP roots are fused at the apex

**Table 1:** Distribution of root number (percent) in MFM (maxillary first molars) and MSM (maxillary second molars)

**Root morphology**	**MFM: N (%)**	**MSM: N (%)**
**Four separate roots**	0 (0)	1 (0.8)
**Four roots (two fused)**	2 (1.6)	1 (0.8)
**Three separate roots**	122 (97.6)	112 (89.6)
**Three roots (two fused)**	1 (0.8)	9 (7.2)
**Three fused roots**	0 (0)	1 (0.8)
**Two separate roots**	0 (0)	1 (0.8)
**Two fused roots**	0 (0)	0 (0)
**Single root**	0 (0)	0 (0)
**Total**	125	125

## Methods and Materials

A total of 250 extracted maxillary molars (125 first molars and 125 second molars) were collected from five metropolises of five geographical areas of Iran: Tehran in the North, Mashhad in the East, Tabriz in the West, Bandar Abbas in the South and Isfahan in the center. The process of sample collection was performed by a team of practitioners who knew the aims of the study, and collection of every tooth was accompanied by a case record stating and confirming the ethnicity of the patients. Debridement was performed after extraction and the teeth were stored in distilled water with 0.5% chloramine until the start of the procedure.

Considering the morphology of the crown being checked by an experienced specialist in operative dentistry and the records given by the practitioners, the teeth were assigned into two groups: maxillary first (*n*=125) and maxillary second (*n*=125) molars.

Every 6 teeth were mounted into foam arches in close contact to each other and an acrylic facing was placed on the facial side to simulate the soft tissue on the radiographs. All the teeth were scanned by a CBCT scanner (ProMax 3D MAX, Planmeca OY, Helsinki, Finland), with a resolution (pixel size) of 160 µm, and a 5×8×8 cm^3^ field of view (FOV). Serial axial, coronal, and sagittal CBCT images were evaluated continuously by moving the toolbar from the floor of the pulp chamber to the apex. Two endodontists analyzed the CBCT scans to investigate the following: number of roots, number of root canals in each root and Vertucci’s root canal classification [19]. Any root canal morphology not conforming to this classification was also recorded. The total number of roots and root canals, the root canal configuration, and the incidence of each variation were analyzed. Inter-rater agreement was measured between the observers. Intra-rater agreement was measured by having the endodontists re-evaluate one half of the CBCT images in two separate sessions.

## Results

The kappa value for the consensus agreement was 0.815.

***Number of roots***

**Figure 2:**
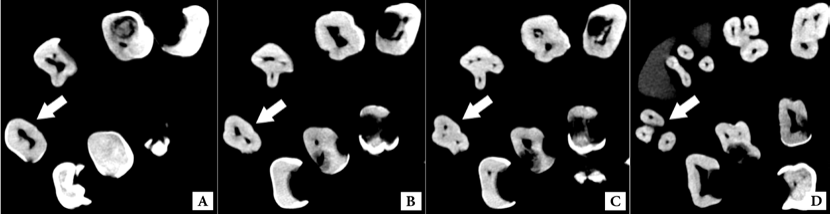
Maxillary second molar with fusion of three roots in the axial section; *A)* White arrow indicates the pulp chamber floor of the examined tooth; *B)* Two canals are seen in this view (B and P): MB and DB are merged; *C)* Three canals (MB, DB and P), DB is separated; *D)* Three separate roots at the apex

The number of roots in maxillary first and second molars is presented in Table 1 in detail. Two maxillary first molars had four roots: one with fusion of the mesiobuccal (MB) and mesiopalatal (MP) roots and the other with fusion of distobuccal (DB) and distopalatal (DP) (Figure 1) (1.6%). Two maxillary second molars had four roots; one with four separate roots and one with fusion of MB and MP roots (1.6%). The most common root morphology was three separate roots in both groups (97.6% in maxillary first molars and 89.6% in maxillary second molars). One maxillary second molar had three fused roots (0.8%) (Figure 2) and one maxillary second molar had two separate roots (0.8%), whereas no maxillary first molar had these anatomic configurations.

***Maxillary first molars:*** The most common canal configuration was Vertucci’s type I in all of the roots [mesiobuccal (MB), distobuccal (DB) and palatal (P)]. The MB roots were conforming to all of the Vertucci’s root canal classification except for type VIII; the most common type was type I followed by types VI, II, and III, in descending order. One additional canal type was 1-3-2-1. The DB root had types I, III and type II configuration, in order of prevalence. For the P root, only two canals represented with canal types other than type I; which were one type II and one type IV configuration. In one maxillary first molar, the MB and DB canals were merged.

***Maxillary second molars:*** The most common canal configuration was type I in all of the roots (MB, DB and P). The DB root had only type I except for one case with type III. The palatal root had only type I. Five maxillary second molars with fusion of two roots had type I canal configuration in MB and DB roots. In four maxillary second molars with fusion of two roots, the MB and DB canals were merged as follows: two separate MB and DB canals, then merging and separating finally (2-1-2), one orifice and then separating into two canals as one MB and one DB (1-2), MB and DB were merged and then separated as 3 and 4 canals (2-1-3-4), one orifice, then separating as 3 canals and finally merging as one apical foramen (1-3-1).

***Configuration of root canal systems***

Inter-rater and intra-rater agreements were 100%. The results of the evaluations of the root canal systems are shown in Table 2. Canal configuration in maxillary second molar with fusion of three roots was: one orifice at the pulp chamber, two canals at the coronal one third (B and P), then DB was separate from MB and finally three apical foramina were seen (1-2-3). This tooth had three separate roots at the apex (Figure 2).

## Discussion

In the present study, the root and canal morphology of maxillary first and second molars were investigated in Iranian population using CBCT technique.

Proper cleaning and obturation of the root canal system is a prerequisite for the success of endodontic treatment. The pulp canal system is complex, and the canals may branch, divide and rejoin. Weine *et al.* [20] categorized the root canal system into four basic types. Others found a much more complex canal system; Vertucci [19] identified eight canal space configurations.

During the last years, CBCT scans have become available for the private dental offices as a diagnostic and treatment planning technique [21]. The main uses of this technology in endodontics are: evaluation of endodontic and non-endodontic pathoses, assessment of root canal morphology, canal preparation, obturation, and removal of root fillings [18, 22], preoperative planning and analysis of internal and external root resorption [23]. Reuben *et al*. [24] reported that CBCT was as accurate as the modified canal staining and clearing technique in identifying root canal morphology. However, CBCT has limited resolution to reproduce detailed root canal morphology. For this reason, micro-CT (μ CT) was introduced for assessment of root canal morphology [25]. Recently two studies concluded that CBCT scanning was a reliable method to detect the second mesiobuccal canal in human maxillary first molars [25, 26].

In two studies by Ng *et al.* [5] and Alavi *et al.* [7], 100% of maxillary first and second molars of the Burmese and Thai populations, had three roots. Al Shalabi *et al*. [27] reported that 97.6% of maxillary first molars in an Irish population had three roots and 2.4% had two roots. In Indian population, 96.8% of maxillary first molars and 93.1% of maxillary second molars had three roots. Our findings are to some extend consistent with those in Irish and Indian populations. In the present study, 97.6% of maxillary first molars had three roots, 1.6% had four roots and 0.8% had two roots. In addition, 96.8% of maxillary second molars had three roots, 1.6% had four roots and 1.6% had two roots.

Prevalence of root fusion in maxillary molars in a Chinese population was 40.1% [28]; whereas our results indicated a lower prevalence (2.4% in maxillary first and 8.8% in maxillary second molars). In the present study the roots were classified as fused when fusion occurred on the entire root surface, but in the aforementioned study, fusion of one-third or less of the roots was regarded as fused roots. The criteria used for designation of fused roots need to be clarified so that it would be possible to decide whether the differences discussed above are true variations or not.

C-shaped roots were reported in two studies of Chinese [28] and Caucasian teeth [29], but they were not found in the present study. In this study, CBCT scans showed that maxillary first permanent molars had one canal (type I) in 46.4% of the MB roots, so more than 50% of MB canals in maxillary first molars had more than one canal.

The most commonly missed canals are the second canals in the MB root [30]. Therefore, to treat or retreat maxillary first permanent molars, dentists need to be aware of the possible existence of two or more root canals before they initiate endodontic treatment. Nevertheless, canal systems like types II and

IV facilitate chemical debridement during canal instrumentation [31]. The number of apical foramina and inter canal communications has importance in surgical endodontics during the process of root-end resection and root-end cavity preparation.

**Table 2:** Configuration of root canal systems in maxillary first and second molars

**Root **	**Type I (1)**	**Type II (2-1)**	**Type III (1-2-1)**	**Type IV (2-2)**	**Type V (1-2)**	**Type VI (2-1-2)**	**Type VII (1-2-1-2)**	**Type VIII (3-3)**
**Maxillary first molars N (%)**								
**MB**	58 (46.4)	18 (14.4)	12 (9.6)	4 (3.2)	2 (0.4)	22 (17.6)	7 (5.6)	-
**DB**	120 (96)	1 (0.8)	3 (2.4)	-	-	-	-	-
**P**	123 (98.4)	1 (0.8)	-	1 (0.8)	-	-	-	-
**Maxillary second molars N (%)**								
**MB**	101 (80.8)	3 (2.4)	5 (4)	4 (3.2)	2 (1.6)	3 (2.4)	2 (1.6)	-
**DB**	119 (95.2)	-	1 (0.8)	-	-	-	-	-
**P**	124 (99.2)	-	-	-	-	-	-	-

*MB, Mesiobuccal; DB, Distobuccal; P, Palatal*

In the present study maximum variations in canal anatomy were discovered in the MB canal of both first and second molars, which was consistent with previous studies [7, 32]. In Thai, Indian and Japanese populations, the most prevalent canal systems in the MB roots were Vertucci’s type I and type IV [7, 20, 31], but in this investigation type I was much more prevalent than type IV (46.4% versus 3.2%) like the Han nation in Chinese population (66.7% versus 8.9%) [4]. In addition, the results of this study showed that type VI was more common than type IV. In the Caucasian population, type II was the most prevalent type [33]. In a study by Kim *et al*. [34] the most predominant canal configuration was type IV. Thirty-three (29.2%) and 20 (17.7%) MB roots had non-classifiable configuration types that could not be classified by either Weine’s or Vertucci’s classification. They used micro-CT in their evaluation, which might be the cause of high occurrence rate of non-classifiable types.

In this study one extra configuration type (1-3-2-1) was first reported in MB root of maxillary first molars and two additional configurations (types 2-1-3-4, and 1-3-1) were first reported in MB root of maxillary second molars. Although this finding contributes to the small percentage of assessed teeth, it is noteworthy in the clinical scenario. Regarding the DB and P root canal system, it should be pointed out that in almost all studies, type I has been reported to be the most prevalent [4, 5, 7, 31, 32]. The incidence of type II in the DB root in Chinese population is remarkable (6.7%) [4]. Although the occurrence rates were low, other rare anatomic types, such as additional canals in DB or P roots, were also reported.

The differences observed between these studies can be attributed to ethnicity, sample size, study protocols (*in vivo* or *in vitro*) and the techniques used to identify canal configuration. Non-invasive CBCT technique shows a higher incidence of anatomic variations compared to previous studies because it facilitates 3D evaluation in larger sample sizes.

There are some previous case reports in Iranian population about extra canals in DB and P roots of maxillary first and second molars which were not observed in the present study. For example: Ghoddusi *et al.* [35] reported two symmetrical maxillary first molars with two separated canals (type II) in their DB roots, Shojaeian *et al.* [36] reported a maxillary second molar with two P root canals and Shakouie *et al.* [37] reported a case series of two maxillary first molars with two separates roots and two root canals.

Rare anatomic variations have not been discovered in this study but they have been reported in some case studies on native Iranians [35-37]. The present study yielded valuable information in the field of endodontics because prior knowledge of anatomic variations leads to effective root canal treatment. Conducting other studies to evaluate the effect of gender, tooth position (right or left side) and age on the incidence of additional canals or the concurrency pattern of additional canals in two bilateral molars with a larger sample size is recommended.

## Conclusion

In a sample Iranian population; *i)* the most common root morphology in maxillary first and second molars was three separate roots, *ii)* maximum variations in canal anatomy were discovered in the MB canal of both groups, *iii)* nearly half of the maxillary first molars had more than one canal in the MB root and *iv)* some rare morphologic variations in this study have not been described except in case reports.
